# Targeted knockout of GABA-A receptor gamma 2 subunit provokes transient light-induced reflex seizures in zebrafish larvae

**DOI:** 10.1242/dmm.040782

**Published:** 2019-11-11

**Authors:** Meijiang Liao, Uday Kundap, Richard E. Rosch, Dominic R. W. Burrows, Martin P. Meyer, Bouchra Ouled Amar Bencheikh, Patrick Cossette, Éric Samarut

**Affiliations:** 1 Research Center of the University of Montreal Hospital Center (CRCHUM), Department of Neurosciences, Université de Montréal, Montréal, QC H2X 0A9, Canada; 2 Department for Developmental Neurobiology, MRC Centre for Neurodevelopmental Disorders, Institute of Psychiatry, Psychology and Neuroscience, King's College London, London SE1 1UL, UK; 3 Department of Bioengineering, University of Pennsylvania, Philadelphia, PA 19104, USA; 4 Department of Paediatric Neurology, Great Ormond Street Hospital for Children NHS Foundation Trust, London WC1N 3JH, UK; 5 Department for Developmental Neurobiology, Centre for Developmental Neurobiology, Institute of Psychiatry, Psychology and Neuroscience, King's College London, London SE1 1UL, UK; 6 Montreal Neurological Institute and Hospital, McGill University, Montréal, QC H3A 2B4, Canada; 7 Neuroscience Department, Centre de Recherche, Centre Hospitalier de l'Université de Montréal, Montréal, QC H2X 0A9, Canada; 8 Modelis Inc., Montréal, QC H2X 0A9, Canada

**Keywords:** Epilepsy, GABA, GABA receptor, Neurological

## Abstract

Epilepsy is a common primary neurological disorder characterized by the chronic tendency of a patient to experience epileptic seizures, which are abnormal body movements or cognitive states that result from excessive, hypersynchronous brain activity. Epilepsy has been found to have numerous etiologies and, although about two-thirds of epilepsies were classically considered idiopathic, the majority of those are now believed to be of genetic origin. Mutations in genes involved in gamma-aminobutyric acid (GABA)-mediated inhibitory neurotransmission have been associated with a broad range of epilepsy syndromes. Mutations in the GABA-A receptor gamma 2 subunit gene (*GABRG2*), for example, have been associated with absence epilepsy and febrile seizures in humans. Several rodent models of *GABRG2* loss of function depict clinical features of the disease; however, alternative genetic models more amenable for the study of ictogenesis and for high-throughput screening purposes are still needed. In this context, we generated a *gabrg2* knockout (KO) zebrafish model (which we called R23X) that displayed light/dark-induced reflex seizures. Through high-resolution *in vivo* calcium imaging of the brain, we showed that this phenotype is associated with widespread increases in neuronal activity that can be effectively alleviated by the anti-epileptic drug valproic acid. Moreover, these seizures only occur at the larval stages but disappear after 1 week of age. Interestingly, our whole-transcriptome analysis showed that *gabrg2* KO does not alter the expression of genes in the larval brain. As a result, the *gabrg2*^−/−^ zebrafish is a novel *in vivo* genetic model of early epilepsies that opens new doors to investigate ictogenesis and for further drug-screening assays.

## Abstract (precis)

**Summary:** The authors present a novel *in vivo* genetic model of idiopathic epilepsy in zebrafish (*gabrg2*^−/−^) to aid the study of ictogenesis and provide a convenient genetic tool for drug screening.

## INTRODUCTION

Epilepsy is a neurological disorder with abnormal, hypersynchronous electrical brain activity causing abnormal movements or cognitive states (Paudel et al., 2019; Vezzani et al., 2016). Epilepsy affects more than 50 million individuals all over the world, causing mortality, disability, social and behavioral stigma (Moshé et al., 2015). Although more than 25 anti-epileptic drugs (AEDs) are available to patients, treatment response is often unpredictable, and approximately one-third of patients fail to gain complete seizure control with pharmacotherapy alone (Chen et al., 2018). Epilepsy can arise from a range of causes associated with different prognoses and treatment outcomes, including metabolic disorders, brain lesions and autoimmune causes, as well as ‘idiopathic’ cases (Sirven, 2015; Thomas and Berkovic, 2014). However, both in: (1) severe epilepsies of early childhood and infancy (McTague et al., 2016) [in particular the early infantile epileptic encephalopathies (EIEEs)], and (2) ‘idiopathic’ generalized epilepsy, the role of genetic mutations is now increasingly recognized (Gardiner, 2005). Consistently, in a recent update of the nomenclature, the ‘idiopathic’ generalized epilepsy syndromes have even been relabeled genetic generalized epilepsies (GGEs). Neurotransmitters are the chemical agents that carry signals across the synapses in the brain and gamma-aminobutyric acid (GABA) is the main inhibitory neurotransmitter in the brain (Olsen and Li, 2012). It binds to GABA-A (GABA_A_) receptors, which act as inhibitory ligand-gated ion channels consisting of five subunits surrounding a central chloride ion pore (Macdonald and Olsen, 1994; Miller et al., 2017; Olsen and Sieghart, 2008; Sigel and Steinmann, 2012). The majority of GABA_A_ receptors in the brain are composed of two alpha (α) subunits, two beta (β) subunits and one gamma (γ) or delta (δ) subunit (Baumann et al., 2002), although diverse stoichiometries have also been reported *in vitro* (Patel et al., 2014). Both GGEs and EIEEs have been linked with mutations in genes encoding GABA_A_ receptor subunits, including *GABRA1*, *GABRB3*, *GABRG2* and *GABRD* (Butler et al., 2018; Cossette et al., 2012; Hirose, 2014; Macdonald et al., 2010, 2006; Moller et al., 2017; Reid et al., 2013; Shen et al., 2017; Sperk et al., 2004)*.*

In particular, numerous mutations in the *GABRG2* gene have been reported to cause epilepsy, about half of which interrupt normal protein translation (i.e. nonsense, splice-site or genomic deletion) (Boillot et al., 2015). Several missense mutations were also reported and are expected to disrupt normal GABA-receptor function by reducing its hyperpolarizing effect. Mutations in *GABRG2* have been most strongly associated with childhood absence epilepsy (a GGE syndrome) and febrile seizures (Baulac et al., 2001; Wallace et al., 2001). Further studies on larger cohorts of epileptic patients confirmed an association of *GABRG2* polymorphisms with GGE more broadly (Chou et al., 2007). Some studies demonstrated *in vitro* that *GABRG2* mutations cause a range of functional defects, RNA alteration, defects in the stability of the resultant protein, trafficking abnormalities, and kinetic defects of the ion channel (Huang et al., 2012; Johnston et al., 2014; Kang et al., 2015). Recently, several mouse models of *GABRG2* loss of function have been generated. In particular, a heterozygous knock-in (KI) mouse model bearing the *GABRG2* Q390X mutation has been generated, and displays reduced cortical inhibition associated with epilepsy phenotypes (Kang et al., 2015; Reid et al., 2013; Tan et al., 2007). The comparison of two mouse models with *GABRG2* loss-of-function mutations [*GABRG2*^+/Q390X^ KI and *GABRG2*^+/−^ knockout (KO)] highlighted different severity in seizure intensity and memory defects. While KI mice exhibited severe seizures and behavioral comorbidities as well as neurodegeneration, *GABRG2*-KO mice display mild absence epilepsy caused by functional haploinsufficiency (Warner et al., 2016). These examples illustrate the complexity of phenotype-genotype relationships in animal models of epileptic syndromes associated with different mutations within the same gene.

In the last decade, zebrafish has emerged as a leading model to investigate biological questions in development and physiology, particularly to decipher basic developmental mechanisms of the central nervous system (CNS) (Blader and Strahle, 2000; Schmidt et al., 2013). One reason for the popularity of zebrafish is its external fertilization, as it makes the embryo suitable for observations from the earliest stages of development. Moreover, this is very convenient for genetic manipulation from the very first cell stage, in particular for CRISPR/Cas9 genome editing (Hwang et al., 2013). Furthermore, the transparency of the embryo allows one to follow *in vivo* organogenesis, in particular the observation of CNS structures with a single-cell resolution. As a vertebrate, zebrafish has a high homology to the human genome, with over 80% conservation of disease-causing genes (Howe et al., 2013), and a complex yet easily accessible CNS, which makes it relevant for the study of human diseases. Indeed, many CNS-related disorders have been successfully modeled in the past, such as amyotrophic lateral sclerosis (Patten et al., 2017), hereditary spastic paraplegia (Gan-Or et al., 2016), autism spectrum disorder (Liu et al., 2018; Marin-Valencia et al., 2018) and epilepsy (Baraban et al., 2013). In particular, zebrafish epilepsy models reproduce several phenotypic features of the disorders and are also very favorable for drug discovery since they can be used in a high-throughput fashion as many early motor assays give easily interpretable readouts for evaluating drug efficacy (Stewart et al., 2014). A recent review showed that zebrafish models of epilepsy are more reliable in terms of clinical relevance and pharmacological features than their mammalian counterparts (Griffin et al., 2018).

Thus, in this study we aimed to establish and characterize a new *in vivo* genetic model of epilepsy caused by mutations in the *GABRG2* gene. We generated a novel *gabrg2*-KO zebrafish model and showed that homozygous larvae undergo light/dark-reflex seizures. We confirmed the neuronal basis for the observed hyperactivity by monitoring calcium levels *in vivo* after light stimulus. Interestingly, the epileptic phenotype of *gabrg2*^−/−^ larvae was only observed during a specific developmental time window (from day 3 to day 6 post-fertilization). We also confirmed that a canonical AED [valproic acid (VPA)] alleviates seizure activity, thus validating the epileptic nature of the neuronal hyperactivity phenotype. Surprisingly, our transcriptomic analysis of the mutant brain suggests that GABRG2 loss of function does not alter the general brain transcriptome. This study brings a new *in vivo* genetic model of epilepsy that is convenient to further study the mechanisms of epileptogenesis/ictogenesis and for evaluating novel therapeutic agents for this particular genetic epilepsy.

## RESULTS

### Knockout of *gabrg2* by CRISPR/Cas9 leads to a decrease in larval survival

**Fig. 1.**
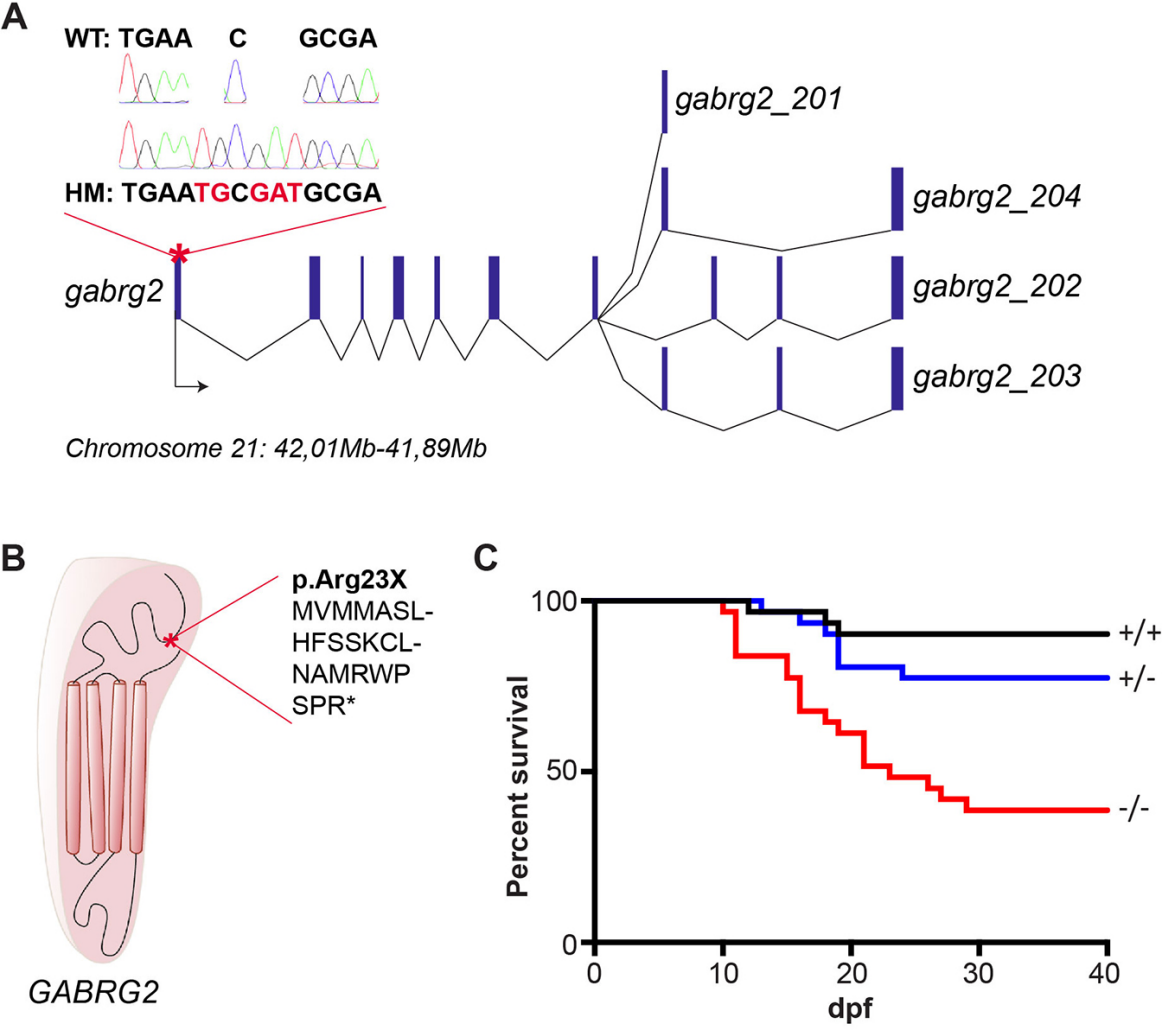
***gabrg2* KO (R23X zebrafish) reduces survival of homozygous larvae.** (A) Scheme of the genomic organization of *gabrg2* zebrafish gene on chromosome 21 with the four transcribed transcripts composed of eight to ten exons. The CRISPR/Cas9-targeted region in exon 1 is indicated by a red asterisk and the +5-nucleotide insertion has been confirmed by Sanger sequencing (chromatograms). WT, wild type; HM, homozygous mutant. (B) Schematic representation of GABRG2 subunit protein domains with the corresponding position of the premature stop codon indicated by a red asterisk. Of note is that the mutation affects the N-terminal GABA-binding domain of the protein, thus preventing the translation of the subsequent transmembrane domains. (C) Survival assays of embryos obtained from an incross between *gabrg2*^+/−^ fish. All three genotypes were determined by high-resolution-melting genotyping at 5 days and the survival of each group was followed for 40 days. Of note is that *gabrg2* KO induces a premature death of *gabrg2*^−/−^ larvae, with only 40% of survival from 30 days post-fertilization (dpf).

The zebrafish genome carries a single ortholog of the human *GABRG2* gene (*gabrg2*: ENSDARG00000053665) and it encodes a protein sharing 82.6% of identity with the human GABRG2 protein (Fig. S1). There are four possible transcripts from the *gabrg2* zebrafish genomic locus on chromosome 2, which are composed of eight to ten exons depending on the alternative splicing events. In order to induce a complete loss of function, we targeted the beginning of exon 1, which is common to all isoforms (Fig. 1A). We successfully isolated a founder fish transmitting a +5-nucleotide insertion (+TG+GAT) that is predicted to lead to a premature stop codon at position 23 of the protein sequence (Fig. 1A,B). This abrogates the translation of the GABA receptor subunit in its N-terminal GABA-binding domain, thus presumably leading to a shortened protein lacking all functional domains. After incrossing heterozygous *gabrg2*^+/−^, we followed the survival of all three genotypes among the offspring [wild type (WT): +/+, heterozygous (HT): +/−, homozygous (HM): −/−] and noticed a decreased survival rate of *gabrg2*^−/−^ larvae specifically from 10 days post-fertilization (dpf) compared to their siblings (Fig. 1C). By 30 dpf, the survival of *gabrg2*^−/−^ is reduced by half, but the remaining survivors are able to reach adulthood and mate. Of note is that there are no particular morphological differences between homozygous mutants and their siblings at any age.

### *Gabrg2^−/−^* zebrafish larvae are hypoactive and display light/dark-reflex seizures

**Fig. 2.**
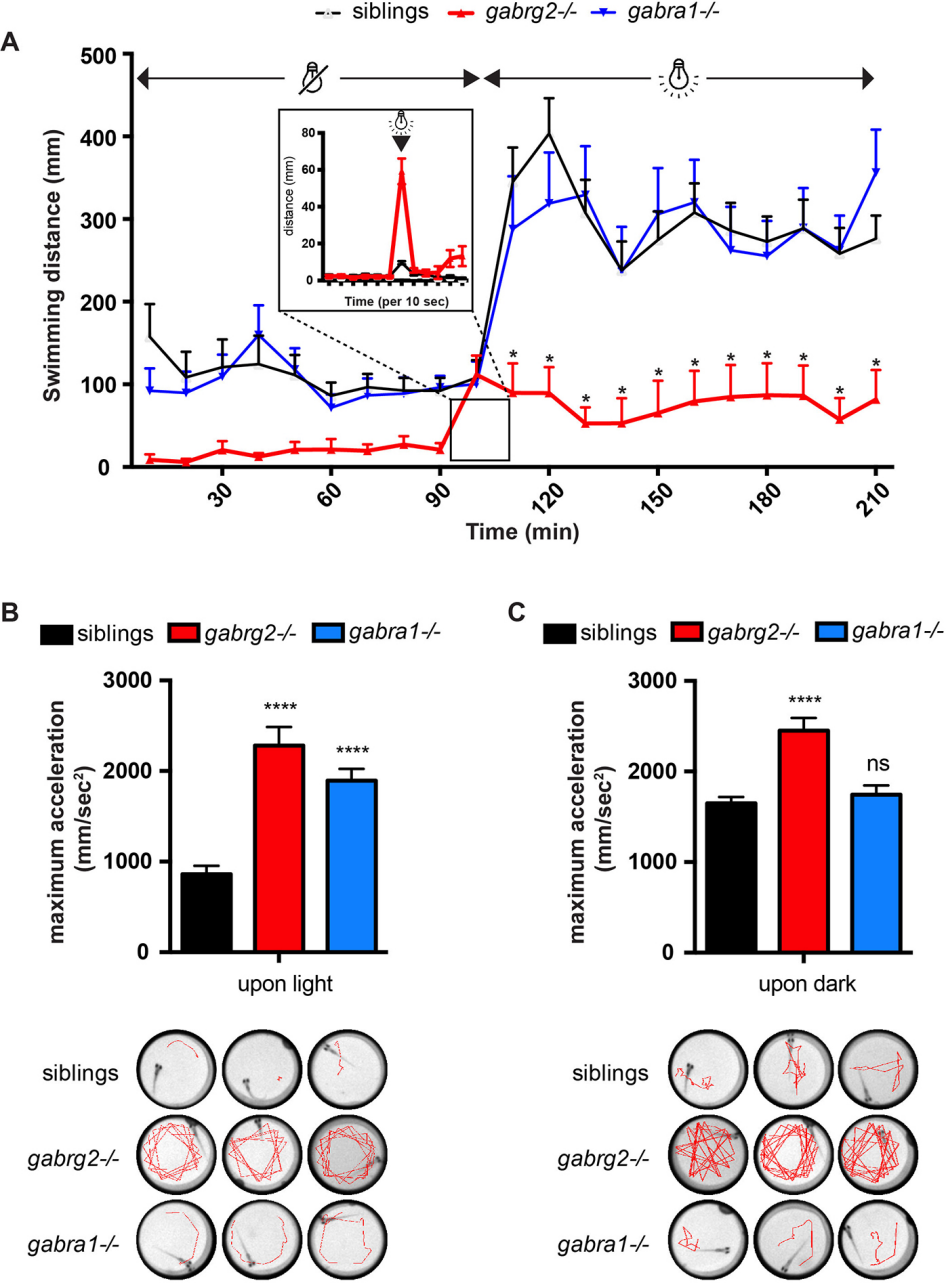
**Light- and dark-triggered hyperactivity in *gabrg2*^−/−^** **larvae.** (A) Four-hour swim assay (2-h dark followed by a 2-h light period) shows that *gabrg2*^−/−^ larvae (5 dpf) are hypoactive compared to their siblings. Interestingly, *gabra1*^−/−^ larvae do not show this hypoactivity phenotype at that stage. (*P<0.05, 2-way ANOVA.) A higher temporal resolution is shown at the light stimulus event. It reveals the sudden hyperactivity of *gabrg2*^−/−^ larvae compared to wild-type siblings. (B) Quantification of the maximum acceleration value over 30 s following light (B) or dark (C) stimuli. The corresponding representative swimming tracks of three larvae per genotype are shown in the lower panel (tracks represent 15 s following photic stimulus). (****P<0.0001, unpaired *t*-test.)

In order to characterize behavioral phenotypes associated with *gabrg2* KO, we monitored the locomotor swimming pattern of 5 dpf *gabrg2*^−/−^ larvae and their siblings during 2-h dark and 2-h light cycles using an automated recording chamber. Interestingly, we noticed a generalized hypoactivity of *gabrg2*^−/−^ larvae compared to their siblings both in dark and light periods, although the difference is only statistically significant during the light phase (Fig. 2A). However, upon light stimulus, we observed that *gabrg2*^−/−^ larvae display a stereotypical whirlpool behavior characterized by uncontrolled movements. This behavior is reminiscent of the increased swimming activity and whole-body convulsions induced by exposure of zebrafish larvae to convulsing drugs (Baraban et al., 2005) (Fig. 2B and Movie 1). Interestingly, we observed the same behavior upon sudden darkness (Fig. 2C). Consistently, we showed that the maximum acceleration of *gabrg2*^−/−^ larvae upon light or dark stimuli is significantly higher than their wild-type siblings (Fig. 2B,C, lower panels). Of note is that *gabrg2*^+/−^ siblings show no behavioral difference compared to their wild-type (+/+) siblings (data not shown).

We previously showed that *gabra1*^−/−^ larvae (mutant for another subunit of GABA receptor) display light-induced brain hyperactivity associated with an increased startling swimming acceleration on light stimulation after a period of darkness (Samarut et al., 2018). Thus, we decided to compare the behavior of *gabrg2*^−/−^ and *gabra1*^−/−^ larvae under the same experimental settings. Remarkably, we observed no difference in the general swimming activity of *gabra1*^−/−^ larvae compared to wild-type siblings, thus suggesting that the hypoactivity observed in *gabrg2*^−/−^ larvae is specific to this genetic condition. Moreover, although in *gabrg2*^−/−^ larvae an increased activity can be triggered by both light or dark stimuli, for *gabra1*^−/−^ larvae this was only observed when switching from dark to light (and not the reverse). Of note is that, at that stage (5 dpf), the hyperactivity phenotype is much stronger in *gabrg2*^−/−^ than in *gabra1*^−/−^ larvae (compare traces in Fig. 2B,C). Moreover, we did not notice any hyperactivity phenotype in response to an increase in temperature (Fig. S2).

Altogether, our results show that *gabrg2*^−/−^ larvae display a stereotypical whirlpool behavior (motor hyperactivity; Movie 1) upon changes in environmental light exposure. The measured hyperactivity is much more pronounced than in *gabra1*^−/−^ larvae of the same age. This phenotype is reminiscent of seizure-like behaviors observed in models of acute seizures in zebrafish larvae upon exposure to a pro-convulsant drug.

### The light/dark-triggered hyperactivity of *gabrg2*^−/−^ larvae is associated with abnormally increased neuronal activity

**Fig. 3.**
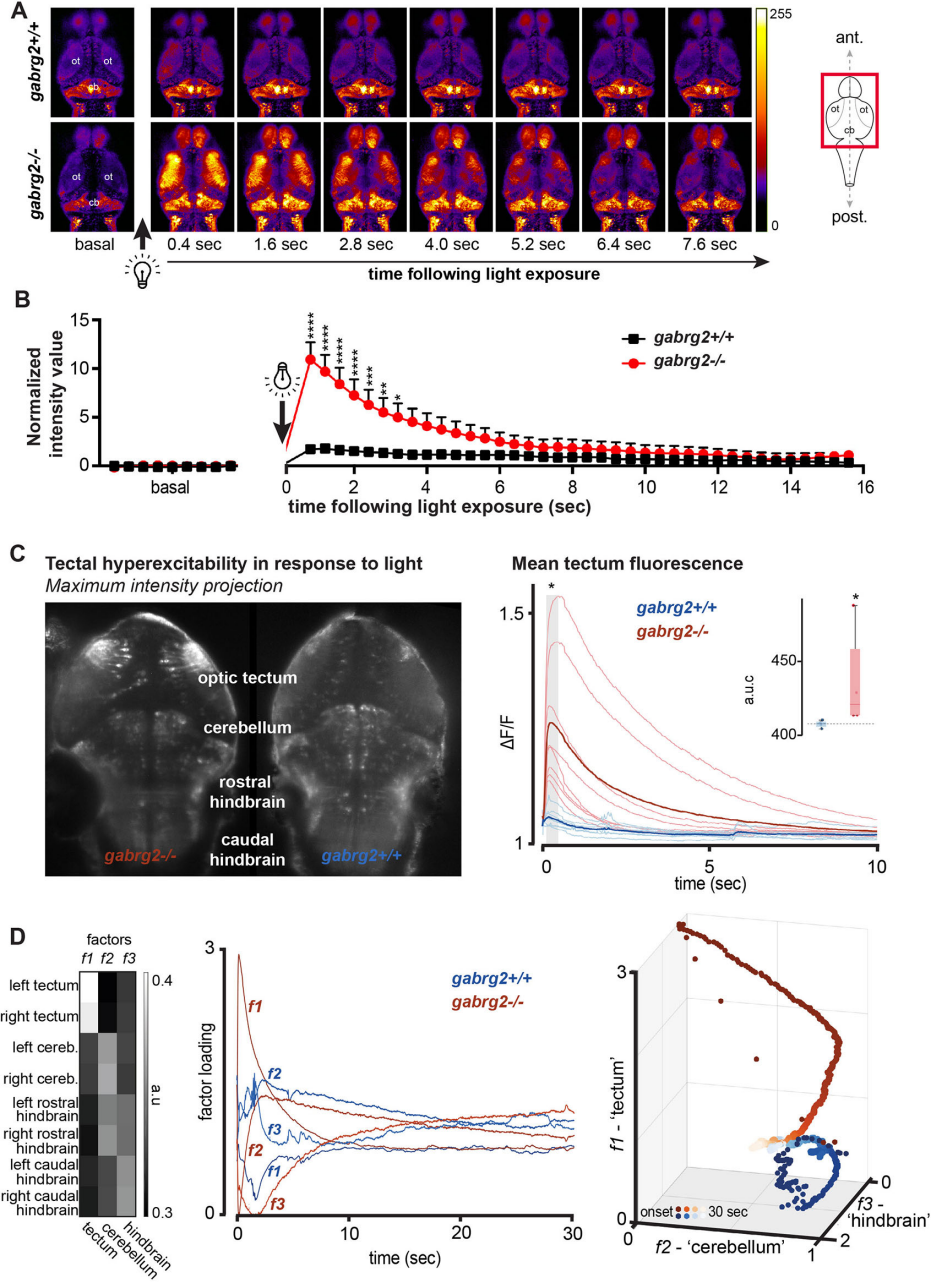
**Calcium imaging reveals broad neuronal activity triggered by light in *gabrg2*^−/−^** **larvae.** (A) *Tg(neurod:GCaMP6f)*^+/−^ and *gabrg2*^+/−^ were intercrossed, and neuronal activity was monitored under a confocal microscope (2.5 Hz) at 5 dpf. After a period of >30 min in complete darkness, switching on the laser induced a broad neuronal activity in both optic tecta in *gabrg2*^−/−^ (*n*=8) compared to *gabrg2*^+/+^ siblings (*n*=7). ot, optic tectum; cb, cerebellum. (B) Fluorescence quantification in the optic tecta before and after laser exposure shows a significant increase of fluorescence in *gabrg2*^−/−^ larvae compared to wild-type siblings. Each point corresponds to the relative quantification of fluorescence from a frame with 400-ms exposure. (****P<0.0001, ***P<0.001, **P<0.01, *P<0.05, two-way ANOVA). (C) Single-plane light-sheet microscopy revealed marked hyperexcitability in the tectum in *gabrg2*^−/−^ fish. The left shows a maximum intensity projection of representative knock-out and wild-type larval fish recordings. The optic tectum shows a marked bilateral increase in fluorescence in the KO, with a much smaller response in the wild-type fish. The right shows Δ*F*/*F* normalized fluorescence intensity for 10 s after switching on visible light, with an early difference in the average amplitude of wild-type and KO tectal fluorescence. Inset shows area under the curve (a.u.c.) of the fluorescence traces from onset to 10 s. (D) A non-negative matrix factorization of regionally averaged fluorescence traces. The factor weights show distinct response patterns for bilateral tectum, bilateral cerebellum/rostral hindbrain and bilateral hindbrain regions (left), with distinct temporal profiles of the loading of these three factors (middle plot). In three-dimensional factorized state-space, there is a clear separation of wild-type and *gabrg2*^−/−^ responses early after being exposed to visible light.

We next wanted to confirm that the observed hyperactivity phenotype has a neuronal correlate. To do so, we measured neuronal activity in *gabrg2*^+/+^ and *gabrg2*^−/−^ larval brains during light exposure using the *Tg(neurod:GCaMP6f)* transgenic line expressing the genetically encoded fluorescent calcium indicator GCaMP6f neuronally (Chen et al., 2013). There was no difference in baseline brain activity of *gabrg2*^−/−^ compared to their siblings (Fig. S3). However, upon sudden exposure to the imaging laser light (following a >30-min period in complete darkness), we observed widely distributed, high-amplitude activity across multiple brain areas, shown by confocal imaging (Fig. 3A) as well as by the quantification of GCaMP6f-driven fluorescence (Fig. 3B). Such a generalized and intense neuronal activity was never observed in *gabrg2*^+/+^ larvae, which display only a modest increase of fluorescence upon laser light stimulation. The generalized brain activity in *gabrg2*^−/−^ larvae lasted several seconds before coming back to basal activity level.

We replicated these findings and further delineated distributed patterns of neuronal hyperexcitability using single-plane light-sheet imaging. This showed particularly high amplitude activity in the optic tectum in *gabrg2*^−/−^ larvae compared to their wild-type siblings, where again none of the wild-type larvae showed levels of fluorescence in the mutant range in the first few seconds after light exposure (Fig. 3C). Using non-negative matrix factorization of regionally averaged fluorescence, we can show that light exposure causes multidimensional changes in fluorescence across multiple brain areas. This reveals a state-space separation of whole-brain activity following light stimulation in *gabrg2*^−/−^ larvae not found in *gabrg2*^+/+^ larvae, indicating abnormal neuronal dynamics compared with wild-type siblings (Fig. 3E).

Altogether, our results suggest that *gabrg2*^−/−^ larvae do not show abnormal basal neuronal activity but undergo a reflex hyperactivity triggered by light stimulus both at the behavioral and neuronal levels.

### Light-induced reflex seizures in *gabrg2*^−/−^ can be alleviated by acute valproic acid exposure

**Fig. 4.**
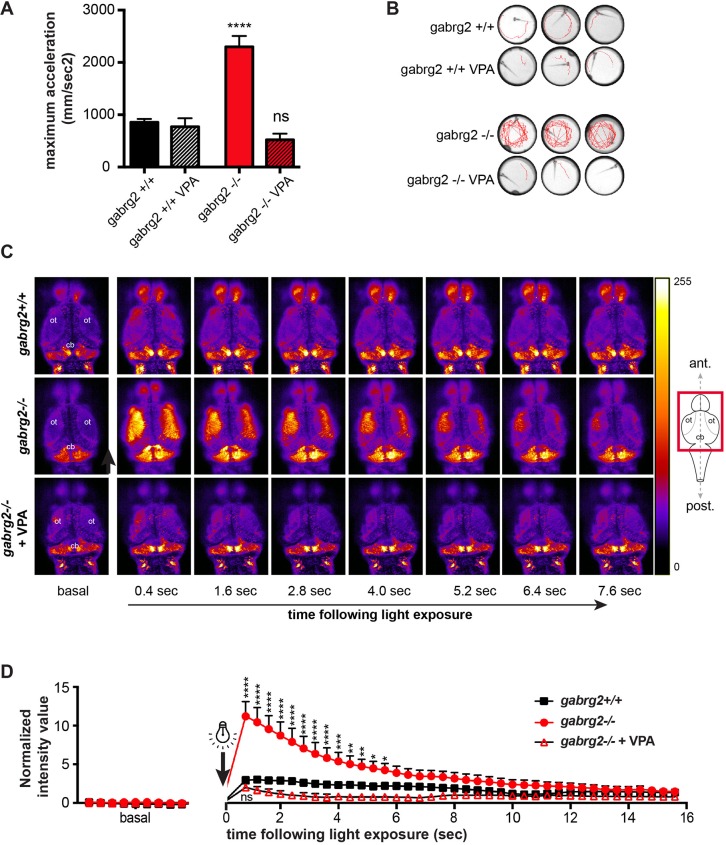
**Valproic acid (VPA) treatment alleviates the movement phenotype and neuronal hyperexcitability of *gabrg2*^−/−^** **larvae.** (A) Quantification of the maximum acceleration upon light stimulus before and after an acute VPA treatment of 200 µM for 2 h, showing the alleviating effect of VPA. (****P<0.0001, unpaired *t*-test.) (B) Representative swimming tracks (15 s following photic stimulus) showing that VPA exposure prevent the whirlpool behavior of *gabrg2*^−/−^ (5 dpf). (C,D) Calcium imaging and fluorescence quantification from *gabrg2*-KO *Tg(neurod:GCaMP6f)* larvae (5 dpf), showing that the neuronal hyperexcitability is relieved after VPA treatment (200 µM for 2 h). ot, optic tectum; cb, cerebellum. (****P<0.0001, ***P<0.001, **P<0.01, *P<0.05, two-way ANOVA).

In order to confirm the seizure nature of the neuronal hyperactivity observed in *gabrg2*^−/−^, we tested the ability of VPA, a canonical AED, to rescue it. Remarkably, we showed that a 2-h incubation with 200 µM VPA completely abolishes the seizure both at the behavioral and neuronal levels (Fig. 4). Indeed, acute VPA exposure completely rescues the maximum acceleration value of *garbg2*^−/−^ larvae to wild-type levels (Fig. 4A). Consistently, we did not observe a whirlpool behavior (e.g. indicative of seizure) upon light in any of the *gabrg2*^−/−^ VPA-treated larvae (Fig. 4B). Although this acute VPA treatment did not induce any lethargy in the larvae, we aimed to confirm that this acute VPA exposure also prevented neuronal firing of *gabrg2*^−/−^ larvae upon light stimulation. By confocal imaging and quantification of GCaMP6f-driven fluorescence from the optic tecta over time, we showed that VPA exposure completely abolishes the hyperexcitability of neuronal activity (Fig. 4C,D).

### Reflex hyperexcitability caused by *gabrg2* KO only occurs at the larval stage

**Fig. 5.**
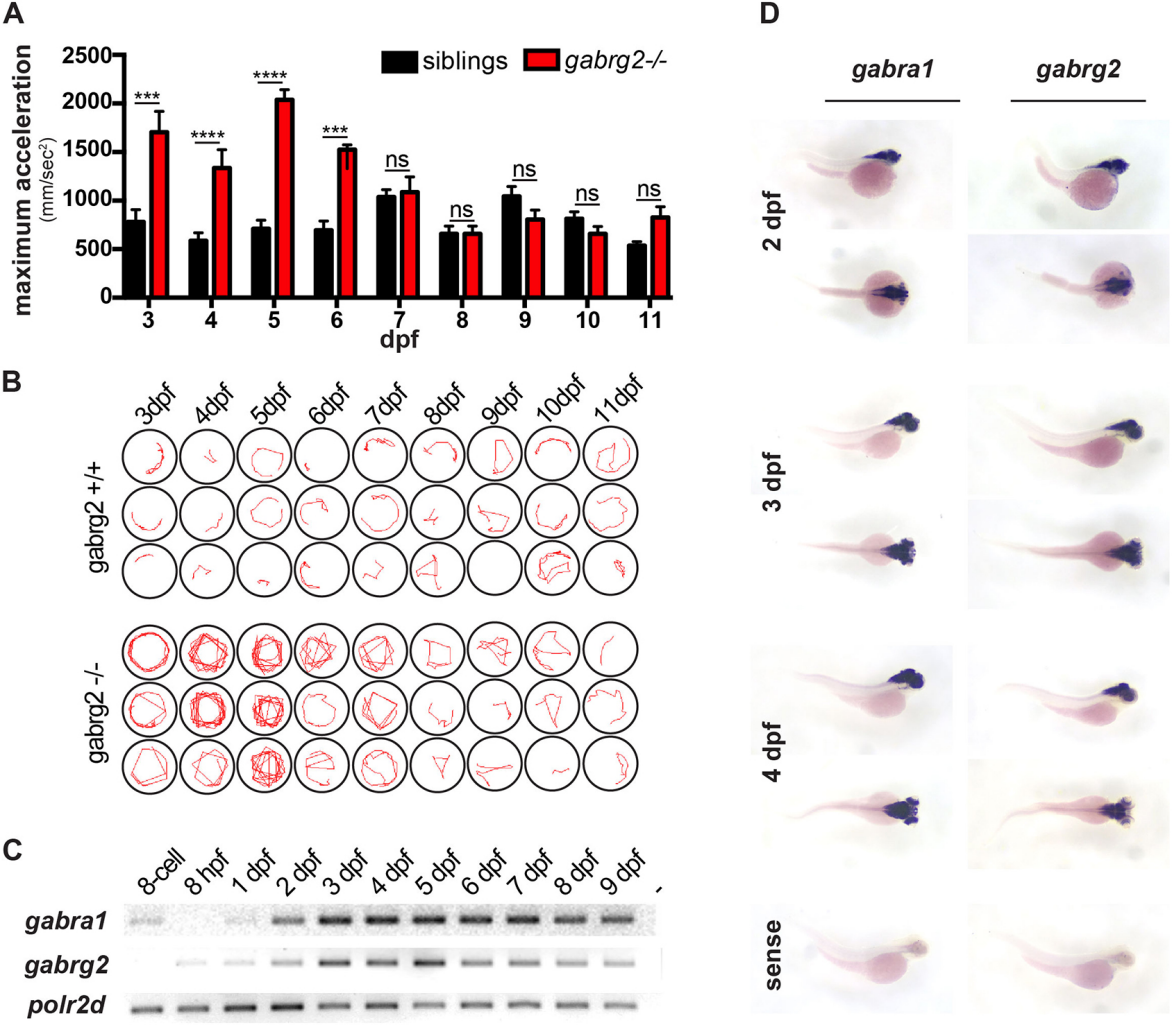
**The photic reflex seizures of *gabrg2***^−/−^ **fish are transient during the early larval stage.** (A) Quantification of the maximum acceleration upon light stimulus of *gabrg2*^−/−^ and *gabrg2*^+/+^ siblings at different ages (from 3 to 11 dpf). A significant difference is observed at 3, 4, 5 and 6 dpf only. (****P<0.0001, ***P<0.001, unpaired *t*-test.) (B) Representative swimming tracks (15 s following photic stimulus) of *gabrg2*^+/+^ versus *gabrg2*^−/−^ larvae at different ages, showing that the stereotypical whirlpool behavior is not observable from 8 dpf. Of note is that the phenotype is the strongest and the most penetrant at 5 dpf. (C) RT-PCR for *gabra1* and *gabrg2* transcripts over wild-type zebrafish development. The *polr2d* gene is used as a house-keeping gene. (D) Whole-mount *in situ* hybridization on whole-mount embryos using an anti-sense probe against *gabra1* or *gabrg2* transcripts. Both genes are strongly expressed in the brain and their expression is largely overlapping. (A sense probe is shown as a negative control.)

So far we have shown that *gabrg2*^−/−^ larvae undergo light- or dark-triggered hyperactivity at 5 dpf. We then aimed to identify the earliest stage at which the embryos are subjected to this reflex phenotype. To do so, we monitored and compared the behavior of *gabrg2*^+/+^ and *gabrg2*^−/−^ larvae in response to light from 3 dpf until 11 dpf (Fig. 5A). Interestingly, we found that maximum acceleration following a light stimulus is already significantly increased at 3 dpf in *gabrg2*^−/−^ compared to their siblings. This is associated with an increased swimming activity leading to a whirlpool behavior reminiscent of seizures (Fig. 5A,B). This hyperactivity upon exposure to a light stimulus is still observed at 4, 5 and 6 dpf but it becomes less penetrant at 7 dpf, with no difference between wild-type and *gabrg2*^−/−^ at 8 dpf. As a matter of fact, it appears that reflex hyperactivity can be triggered by light/dark stimuli in *gabrg2*^−/−^ larvae from 3 to 6 dpf only.

Interestingly, we previously showed that *gabra1*^−/−^ larvae have increased neuronal activity in response to light from 4 dpf, with continually worsening seizure phenotypes until the juvenile stage, eventually becoming lethal after 6 weeks of age (Samarut et al., 2018). In this work, we showed that *gabrg2*^−/−^ larvae undergo a light/dark-triggered seizing phenotype during the larval stage only. Thus, we wondered whether this transient occurrence could be explained by a different temporal expression of GABRA1 and GABRG2 subunits. As a result, we checked expression of *gabra1* and *gabrg2* transcripts during development by reverse-transcriptase polymerase chain reaction (RT-PCR), from the eight-cell stage (at which the zygotic transcription has not yet started) until 9 dpf (Fig. 5C). Interestingly, we found that *gabra1* transcript is detected among the maternal transcripts but starts to be expressed zygotically from 1 dpf with a more robust expression at 2 dpf that is sustained constantly over time from 3 dpf. However, *gabrg2* transcript only starts to be detectable from 8 hours post-fertilization (hpf) and increases over time until 5 dpf. We further note a decrease in the expression of *gabrg2* transcript from 6 dpf, with a weaker expression at 9 dpf (Fig. 5C). As a result, *gabrg2* expression appears to peak at 5 dpf, whilst *gabra1* expression levels are sustained from 3 dpf. We further examined regional expression of both transcripts at larval stages by whole-mount *in situ* hybridization, and observed that they are both strongly expressed at 2, 3 and 4 dpf (Fig. 5D). We showed that, at these stages, both *gabra1* and *gabrg2* transcripts are broadly expressed in the brain and that their regional expression is largely overlapping.

### *Gabrg2* KO does not change the larval brain transcriptome

Since we showed that *gabrg2*^−/−^ larvae undergo reflex hyperexcitability that is reminiscent of seizures at larval stages, we wondered whether these brain hyperactivity events were associated with changes in gene expression. Hence, we conducted a whole-transcriptome sequencing analysis (RNAseq) from whole larval brains extracted from 5 dpf *gabrg2*^+/+^ and *gabrg2*^−/−^ larvae (Fig. S4A). Surprisingly, differential expression analysis only reported that a single gene was significantly upregulated in the brain of *gabrg2*^−/−^ larvae compared to wild-type siblings (Fig. S4B,C). Although there are some differences in the raw expression of certain genes between *gabrg2*^+/+^ and *gabrg2*^−/−^ samples (i.e. *pvalb4*, *nkx6.3*; Table S1), our differential expression analysis shows that these changes are not consistent between triplicates. Therefore, it is impossible to conclude whether these changes are natural variations between samples or whether they can be specifically attributed to the mutation. As a result, the Ras-related associated with diabetes gene (*rrad*: ENSDARG00000052011) was the only gene statistically upregulated by a fold change of 1.36 (logFoldChange 0.44, *P-*value 7.09E^−3^; Table S1). Consistently, sample hierarchizing by clustering and principal component analysis also revealed no clear genotype-specific clustering nor distinguishable patterns between *gabrg2*^+/+^ and *gabrg2*^−/−^ samples (Fig. S4D,E). We also confirmed the quality of cDNA library preparation (Fig. S4F) and that all triplicates of *gabrg2*^+/+^ and *gabrg2*^−/−^ samples that have been sequenced only contained the wild-type or mutant sequence, respectively, thus validating the experimental procedure (Fig. S4G). Moreover, we checked the expression of genes that we previously identified as being dependent on GABA receptor signaling (*gabra1*, *gabrg2*, *gata3*, *kcc2*, *sema6d*, *cacna2d2a*) (Samarut et al., 2018) and confirmed by qRT-PCR that their expression is not significantly affected upon *gabrg2* KO (Table S2). Interestingly, the fact that the expression of the *gabrg2* mutant transcript is not reduced in *gabrg2*^−/−^ larvae suggests that the mutation does not induce degradation of the messenger RNA through nonsense-mediated decay. As a result, it appears that *gabrg2* KO does not lead to obvious differences in the expression of genes within the brain at the larval stage.

## DISCUSSION

In this work, we introduce a novel zebrafish model of a human genetic epilepsy. We show that zebrafish carrying a knockout of the *gabrg2* gene – orthologous to the human epilepsy gene *GABRG2* – show inducible, short-lived whole-body convulsions associated with widespread increases in brain activity. This activity is confined to the larval stages (consistent with the developmental expression profile of the *gabrg2* gene) and, whilst reducing overall survival, *gabrg2*^−/−^ fish can reach adulthood.

The hypermotor phenotype is associated with widespread increases in brain activity and can be rescued with anti-epileptic medication, indicating that the phenotype is epileptic in nature, even though we did not observe spontaneous seizure-like events in these fish (Fig. S3). In human patients, epileptic seizures triggered by specific sensory events, environmental factors or cognitive states are considered ‘reflex seizures’ (Panayiotopoulos, 2005a). Whilst the majority of patients with reflex seizures will also have unprovoked seizures, there is a subgroup of patients with ‘reflex epilepsy’ who only ever experience seizures in response to provoking stimuli (Okudan and Ozkara, 2018). Furthermore, in humans, abnormal brain dynamics in response to photic stimulation (i.e. a photoparoxysmal response on EEG recording) are both highly heritable and are associated with an increased risk of developing epilepsy (Tauer et al., 2005). In fact, case reports identify photosensitivity as a potential phenotypic feature of *GABRG2*-related epilepsy in humans. Our novel *gabrg2*^−/−^ model may therefore offer insights into the genotype-phenotype relationship between abnormal GABA signaling and brain hyperexcitability, which at its extremes causes epileptic seizures like the ones observed in our model.

Surprisingly, although *GABRG2* mutations have often been associated with febrile seizures (Boillot et al., 2015; Hirose, 2014), we did not observe any particular effect of increasing the temperature of the bathing water (up to 31°C for 15 min; Fig. S2). Yet, epileptic seizures can be triggered by transient hyperthermia in zebrafish larvae and have been shown to be evocative of febrile seizures in humans (Hunt et al., 2012). This highlights the fact that particular aspects of the epileptic phenotype could be due to specific mutations in the gene. In other words, some specificity of the epileptic syndrome may be due to the level of haploinsufficiency that is driven by a specific mutation. In this context, a complete loss of function as we induced here might therefore not mimic the broad spectrum of the epileptic syndromes associated with all the mutations in one gene. This emphasizes the idea that epilepsies should not be classified solely based on the affected gene, since different mutations in the same gene could have drastically different effects (i.e. different types of seizures, manifestations or triggers). Thus, our findings confirm the fact that each genetic background associated with epilepsy should be considered uniquely in terms of epileptogenesis, ictogenesis and, therefore, treatment responsiveness.

Another interesting aspect of the phenotype we are describing is the fact that it is limited to the larval stage. Indeed, whilst survival overall is reduced, *gabrg2*^−/−^ fish can reach adulthood and ‘outgrow’ their seizures. In fact, in human patients on the severe end of the phenotypic spectrum of *GABRG2*-related epilepsies, seizures start very early in life. In one recent cohort of EIEE patients, mean age of onset was 7.7 months (range 1 day-12 months) (Shen et al., 2017). Many of the milder phenotypes also start in childhood and, for patients with the most strongly associated clinical phenotypes – childhood absence epilepsy (a GGE syndrome) or febrile seizures (Boillot et al., 2015; Kang and Macdonald, 2016) – seizures typically remit by the end of puberty. In fact, transient epilepsies are the most common epilepsy phenotypes in childhood, largely due to the prevalence of benign childhood epilepsies/seizure susceptibility syndromes (Panayiotopoulos, 1993). Two of the most common human benign epileptic syndromes are rolandic seizures (RS; also known as benign childhood epilepsy with centrotemporal spikes) (Herranz, 2002) and Panayiotopoulos syndrome (PS; also known as early onset benign childhood occipital epilepsy) (Loiseau and Duche, 1992). In RS and PS, seizures start between 1 and 14 years of age, and patients are seizure free after 2-4 years of onset (RS) or 1-2 years of onset (PS) and no longer need AEDs (Panayiotopoulos, 2005b). Thus, about 27% of patients with PS manifest only one seizure in their life (Panayiotopoulos, 2005b). At this juncture, our novel line therefore captures further aspects of the natural history of the human disease. Indeed, the transient reflex-seizure phenotype of *gabrg2*^−/−^ fish, confined to the larval stage, is in stark contrast to existing zebrafish models of epilepsy (Griffin et al., 2018; Samarut et al., 2018).

Our results showing that *gabrg2* transcript is maximally expressed at 3, 4 and 5 dpf could explain why *gabrg2*^−/−^ larvae only display the seizure phenotype at these stages. Yet, in the normally developing larvae, *gabrg2* expression is still detectable at later stages (i.e. 8-9 dpf), whilst the hyperactivity phenotype remit at that stage. Taken together with the distinct developmental profile of *gabra1* expression, this observation suggests that the transient time window during which a seizing phenotype can be induced in *gabrg2*^−/−^ larvae reveals a particularly sensitive developmental period. GABA_A_ receptor subunit expression is dynamically regulated throughout the lifespan, resulting in different stoichiometry of GABA_A_ receptor subunit composition at different times of development and/or in different regions of the brain. Thus, the lack of one specific subunit at a specific time at a specific place could lead to hyperexcitability and to an epilepsy phenotype. Yet at a different stage of development that subunit may be less crucial and have less of an observable effect on brain function.

Surprisingly, although we previously showed that *gabra1* KO leads to defects of neurodevelopment, our present work suggests that *gabrg2* KO does not. Indeed, 460 genes were found to be differentially expressed in the brain of 5 dpf *gabra1*^−/−^ mutants (Samarut et al., 2018). Specifically, we showed that development of the inhibitory network was impaired in *gabra1*^−/−^ larvae and that this neurodevelopmental component of the pathology might play an important role in the excitation/inhibition imbalance of the mutant brain throughout lifespan. Our present work shows almost no difference at the transcriptomic level in *gabrg2*^−/−^ mutants compared with wild type, suggesting that, although being expressed at early stages of development, the gamma 2 subunit does not seem to play a crucial role in broader GABA-mediated neurodevelopmental processes, at least at the transcriptomic level. This is consistent with the fact that *gabrg2*^−/−^ fish are only undergoing seizing events at the larval stages and not later in life, and also with the fact that the homozygotes can reach adulthood [unlike *gabra1*^−/−^ (Samarut et al., 2018)]. It is now well admitted that GABA signaling regulates many aspects of neurodevelopment, including synaptogenesis. Interestingly, our present work brings one more piece of complexity by suggesting that the different GABA receptor subunits do not influence the same processes meditated by GABA signaling.

Lastly, our work brings a new *in vivo* genetic model of idiopathic epilepsy that is convenient for a broad range of scientific applications. Indeed, the fact that the hyperactivity phenotype can be triggered by light or dark stimuli greatly improves the standardization of the seizure assays, thus potentially facilitating further drug-screening applications. Moreover, this new genetic model will open new doors of investigation, in particular if studied in parallel to the *gabra1*^−/−^ zebrafish mutant (Samarut et al., 2018). Indeed, such studies would be important to decipher the specific role of each GABA receptor subunit at the cellular, molecular and electrophysiological levels.

## MATERIALS AND METHODS

### Fish husbandry and fish lines

Wild-type *Danio rerio* were raised at 28.5°C, kept under a 12-h dark, 12-h light cycle, and staged as described in Swaminathan et al. (2018). All experiments were performed in compliance with the guidelines of the Canadian Council for Animal Care and conducted at the Centre de Recherche du Centre Hospitalier de l'Université de Montréal (CRCHUM), and approved by the Comité Institutionnel de Protection des Animaux of CRCHUM (approval # N15018PMDz). All experiments were performed on sexually undifferentiated zebrafish larvae. Humane endpoints were in place during the study and all animals were monitored and assessed daily for well-being as per guidelines established by the Canadian Council of Animal Care committee at CRCHUM. Behavioral signs of poor health in adult animals necessitating euthanasia included an inability to feed or swim. Physical abnormalities were also monitored daily and adult animals displaying a distended abdomen, skin ulcerations/wounds and skeletal deformities were euthanized immediately by anesthetic overdose and rapid chilling. The *Tg(neurod:GCaMP6f)* transgenic line was a gift from Claire Wyart (Institut du Cerveau et de la Moelle Epinière, Paris, France).

### Whole-mount *in situ* hybridization and probe cloning

A specific 911-bp *gabrg2* probe was amplified from total RNAs extracted from 1-day-old embryos using the following primers: F 5′-ATTTTTCCTCTAAATGCCTGAACGCGATG-3′; R 5′-CGTTGTGATTCCCAAGGAGGTTCG-3′. The PCR fragment was then cloned within the pCS2+ vector using the TOPO TA cloning kit (Invitrogen). Whole-mount *in situ* hybridization using a *gabra1* probe on zebrafish embryos was performed as described previously (Samarut et al., 2016).

### sgRNA and *Cas9* preparation and microinjection

A gRNA sequence was designed using the online tool CRISPRscan to target the following early coding sequence of the *gabrg2* gene (ENSDART00000075740.4) (protospacer adjacent motif site in parentheses): GAACGCGATGGCCATCCCCG(CGG). Synthesis of gRNA and *Cas9* mRNA as well as embryo microinjection was performed as described previously (Samarut et al., 2016).

### Fish tracking and seizure triggering

A DanioVision (Noldus, Wageningen, The Netherlands) setup was used as a lightproof recording chamber with an infrared camera. Light-induced seizures were induced after 1 h spent in darkness and by switching on the built-in cold-white-light-emitting diode light for at least 1 min. Ethovision XT12 (Noldus) was used for analyzing the distance swam and maximum acceleration as well as to extract swimming tracks.

### Antiepileptic drug treatment

A 1 M solution of VPA (Sigma, St Louis, MO, USA) was prepared extemporaneously in water and was subsequently dissolved in fish water to reach the final concentration.

### GCaMP6f monitoring and fluorescence quantification

For imaging experiments all larvae were raised in Danieau’s solution [69% NaCl_2_+24% HEPES buffer+4% Ca(NO_3_)_2_+2% MgSO_4_+1% KCl pH 7] diluted at 1:50 in deionized water, with 1-phenyly-2-thiourea (PTU) to prevent pigment formation, thus maximizing optical accessibility (Karlsson et al., 2001).

For confocal imaging, 6 dpf larvae were immobilized dorsal in 1% low-melting agarose and mounted dorsal side up under a spinning disk confocal microscope. The embedded fish was kept in complete darkness for at least 30 min. The seizure was triggered by switching on the fluorescence excitation laser. Single-plane images were acquired at 2.5 Hz for 60 s. Fluorescence quantification from the optic tecta was performed using the mean gray value measurement from an 8-bit image (ImageJ, NIH, Bethesda, MD, USA). All gray values were normalized against a resting basal mean gray value.

For light-sheet imaging, non-anesthetized larvae at 6 dpf were immobilized in 2.5% low-melting-point agarose (Sigma-Aldrich) prepared in Danieau’s solution and mounted dorsal side up on a raised glass platform that was placed in a custom-made Danieau’s-filled chamber. The embedded fish was kept in complete darkness for 45 min. The seizure was triggered by switching on the fluorescence excitation laser from a custom light-sheet microscope [microscope details are reported in Rosch et al. (2018)]. Single-plane images were acquired at 40 Hz for 5 min. Gross anatomical regions were segmented manually and regional fluorescence average traces were extracted from the first 30 s of the recording, and normalized to the baseline level achieved during the last 30 s of the 5 min recording window. Here, we report normalized florescence intensity (Δ*F*/*F*) values for the regional averages.

Constrained least-squares non-negative matrix factorization over a *r*×*t* matrix (*r*, number of regions; *t*, time samples; where each entry represents a fluorescence value at the time *t* in region *r*) was run to identify a set of three fluorescent factors to explain the maximum variance in the data.

### RT-PCR

Reverse transcription was performed from 1 µg of total RNA using the superscript VILO reverse transcription mix (Invitrogen). PCR was performed on 1 µl of cDNA using regular GeneDirex Taq polymerase (FroggaBio) using the following gene-specific primers: gabrg2_F: 5′-GGATCAACAAGGATGCAGTG-3′; gabrg2_R: 5′-GAAAAGAGCCGCAGGAGAG-3′; gabra1_F: 5′-TCGAGCCATCCTGATTTTTC-3′; gabra1_R: 5′-TCAGCCTTTCATCCTTCCAG-3′; polr2d_F: 5′-AACGCAAAGTGGGAGATGTG-3′; polr2d_R: 5′-TGTAACTCCTCATCCTCGAACC-3′.

### Transcriptomic assay, differential expression assay and pathway analysis

Briefly, 5 dpf larvae were anesthetized in 0.04 mg/ml tricaine in cold calcium-free Ringer's. The larvae was pinned laterally and the brain was gently extracted manually using Dumont#5 fine forceps. The dissection was performed in cold calcium-free Ringer's. Extracted brains were immediately flash frozen in liquid nitrogen for further extraction. Total RNA was extracted from whole brains of 5 dpf *gabrg2*^−/−^ and *gabrg2*^+/+^ larvae using picopure RNA extraction kit (Thermo Fisher Scientific) following the manufacturer's standard protocol. Quality of total RNA was assessed with the BioAnalyzer Nano (Agilent) and all samples had an RNA integrity number (RIN) above 9. Library preparation and sequencing was done at the Institute for Research in Immunology and Cancer's Platform (University of Montreal) as described by Swaminathan et al. (2018). Sequencing was performed with the Illumina NextSeq500 using the SBS Reagent Kit v3 (80 cycles, paired-end) with 1.6 nM of the pooled library. Cluster density was targeted at around 800k clusters/mm^2^. The quality of the reads from the paired-end 80-bp sequencing on NextSeq500 was correct for all samples with a number of reads between 171 million and 192 million, and an average of 87% of them passing the quality filter. Sequences were trimmed for sequencing adapters and low-quality 3′ bases using Trimmomatic version 0.35 (Bolger et al., 2014) and aligned to the reference genome version GRCz10 (gene annotation from Ensembl version 87) using STAR version 2.5.1b STAR (Dobin et al., 2013). On average, 92% of the reads could be successfully mapped onto the genome using STAR, allowing an average coverage of 130×. Gene expressions were obtained both as readcount directly from STAR as well as computed using RSEM (Li and Dewey, 2011) in order to obtain transcript level expression. Differential gene expression analysis was assessed by DeSeq2 package using R software. Differential gene expression was filtered on false discovery rate (or adjusted *P*-value>0.05).

## Supplementary Material

Supplementary information
