# Supplementary material for: Targeted knockout of GABA-A receptor gamma 2 subunit provokes transient light-induced reflex seizures in zebrafish larvae
Source: Dis Model Mech. 2019 Nov 11;12(11):dmm040782. doi: 10.1242/dmm.040782 (PMC6899022; doi:10.1242/dmm.040782)
Supplement: Supplementary information [file dmm-12-040782-s1.pdf]

Figure S1: Protein sequence alignment between human (*Homo sapiens*) and zebrafish (*Danio rerio*) GABRG2 proteins. The coverage is 99.4% between both sequences with a percentage of identify of 82.6%. (human sequence: AAD50273.1, zebrafish sequence: NP_001243179.1).

PROTEIN SEQUENCE ALIGNEMENT
1- *Homo sapiens* GABRG2: AAD50273.1
2- *Danio rerio* GABRG2: NP_001243179.1

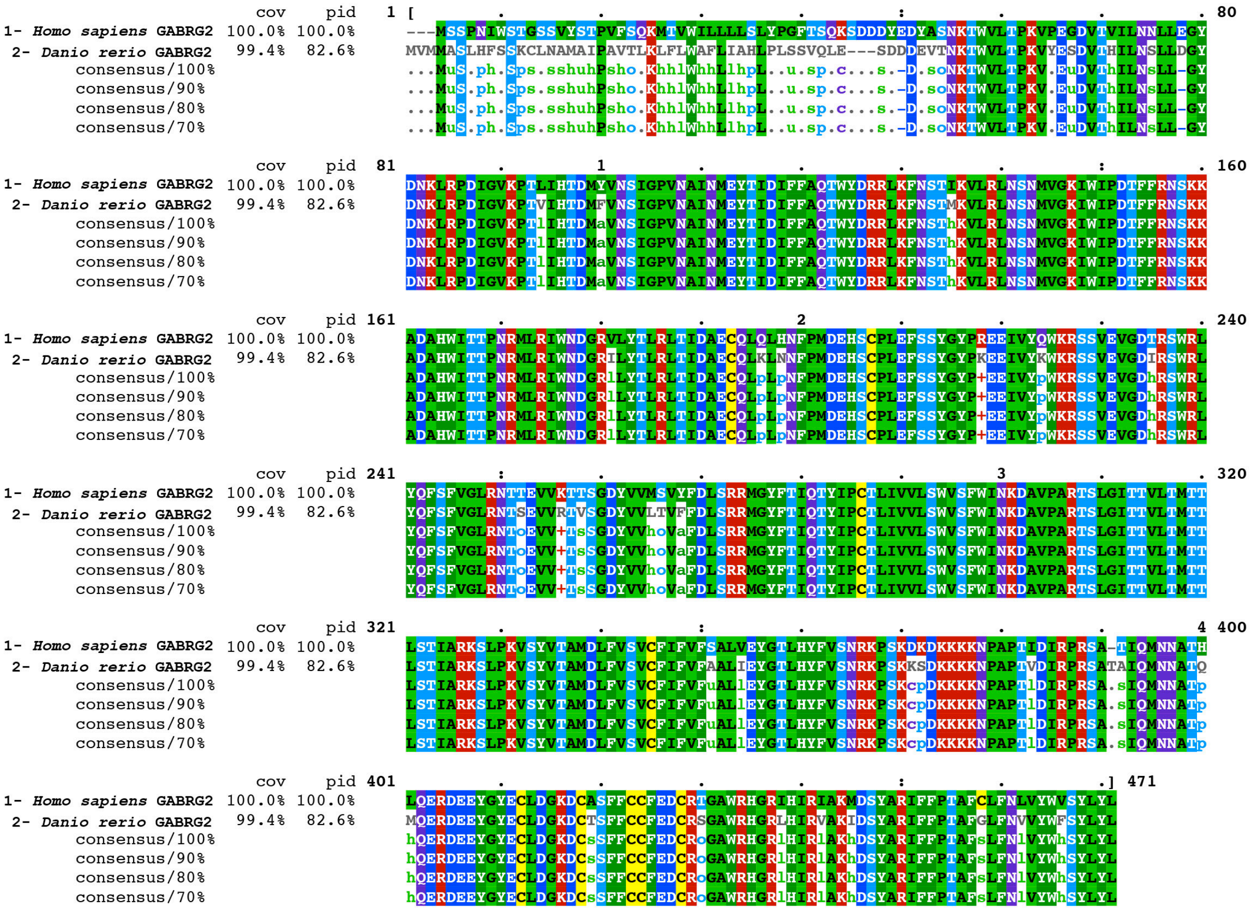

Figure S2: Temperature increase does not induce any specific behavioural changes in gabrg2-/- larvae. (A) Swimming distance and (B) maximum acceleration of 6 dpf gabrg2+/+, +/- and -/- larve have been monitored during a transient temperature increase (+7C over 15 minutes). No specific behaviour is noticed among *gabrg2*-/- population except the general hypoactivity compared to sublings described previously. In particular, we did not notice any drastic increase

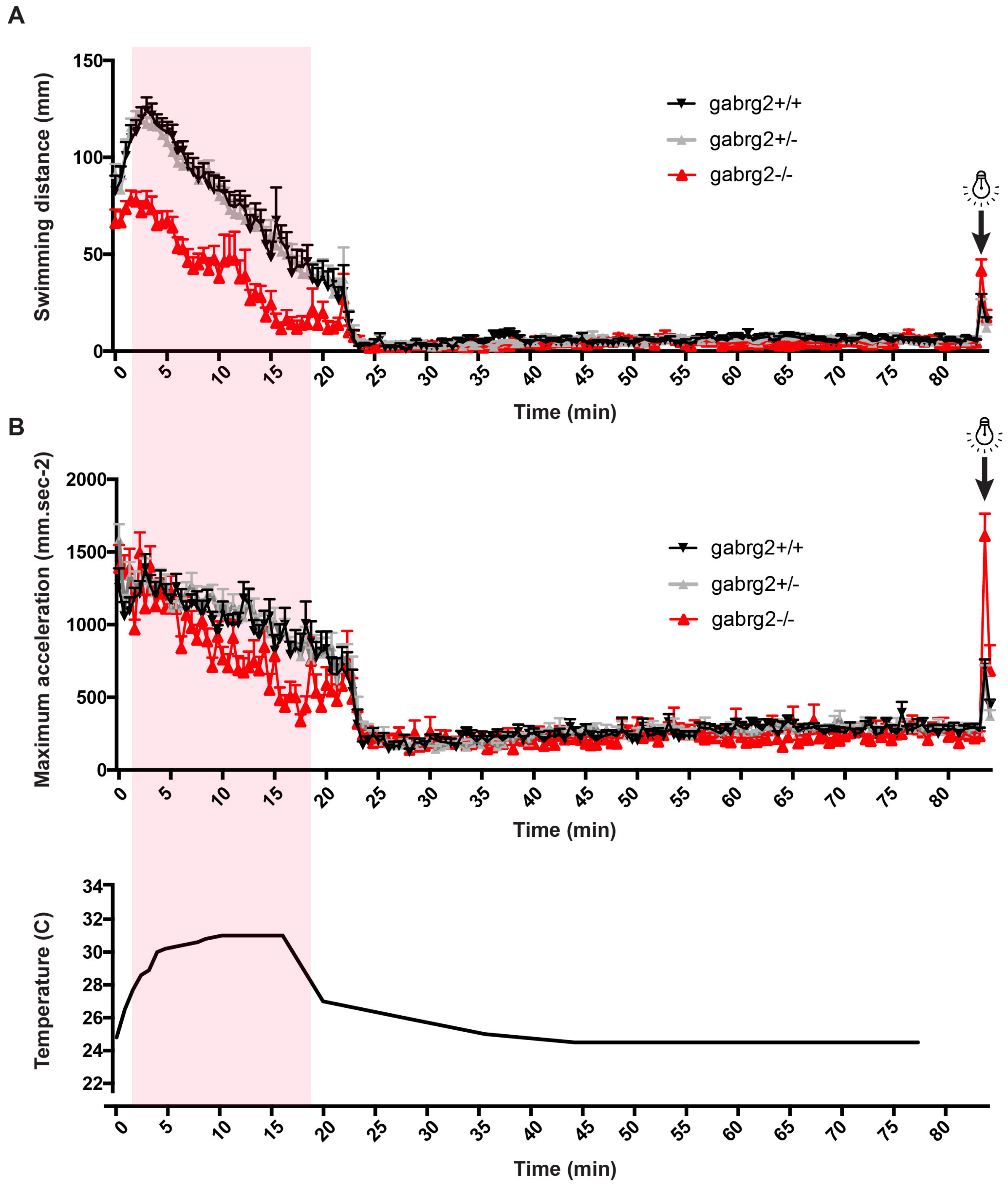

Disease Models & Mechanisms • Supplementary information

of swimming activity that would be informative of seizure as it is the case with a light stimulus (arrow).

Figure S3: No difference of basal neuronal activity between gabrg2+/+ and -/- larvae. *Gabrg2*+/− and [NeuroD:GCaMP6f] +/− were intercrossed, and neuronal activity was monitored under a confocal microscope (2.5 Hz) at 5 dpf. After the neuronal hyperactivity triggered by the LASER stimulus, we recorded the basal activity for 10 minutes.

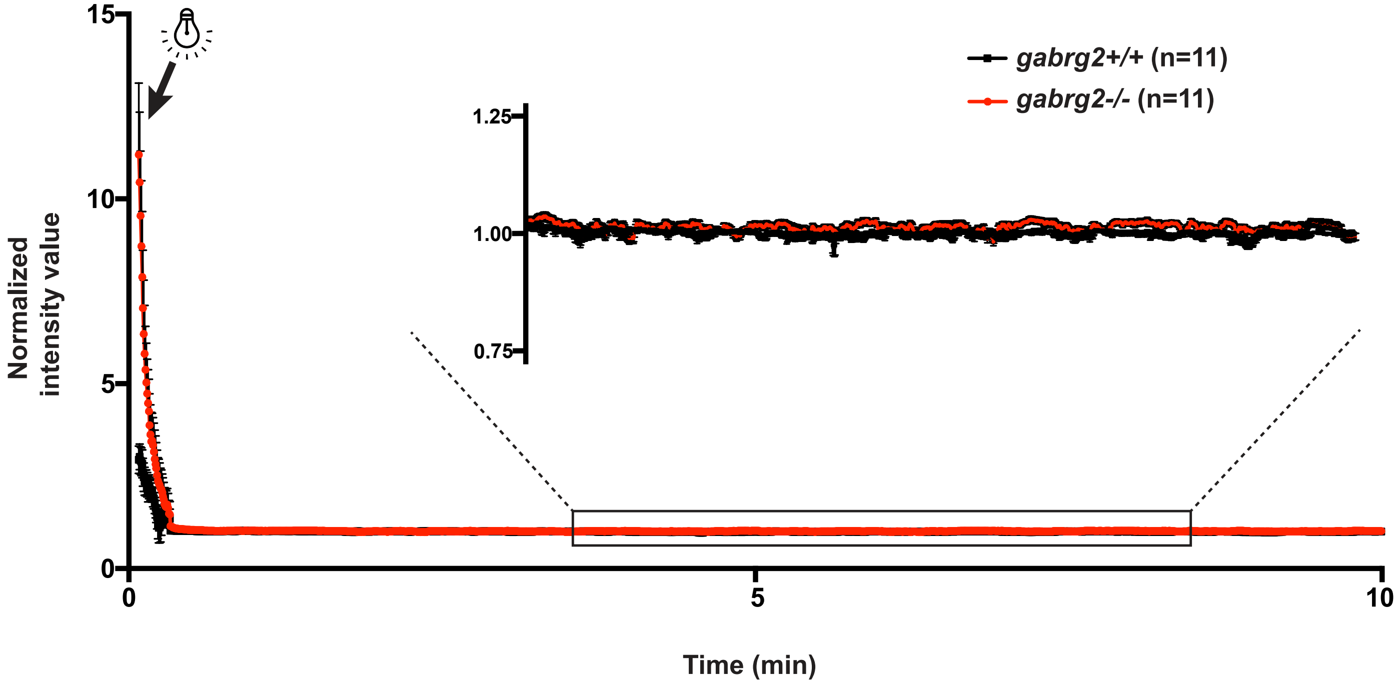

Disease Models & Mechanisms • Supplementary information

Figure S4: Whole transcriptome analysis of gabrg2-/- brains identified no major changes. (A) Experimental outline of the whole brain transcriptomic assay from 5 dpf larvae. (B) Volcano plot and MA plot (C) showing the unique significantly differentially expressed gene (*rrad:* logFoldChange 0.44, pvalue 7.09E^{-3} , Table S1) highlighted in red (arrow). **Complementary clustering analysis of the transcriptomic data show no clustering per genotype.** (D) Principal component analysis displayed for the first two most significant components explaining the variability amon samples. (E) Hierarchy of samples based on normalized log readcounts produces the following. (F) Validation of cDNA library preparation and hit coverage of WT and mutant gabrg2 transcript in sequences samples.

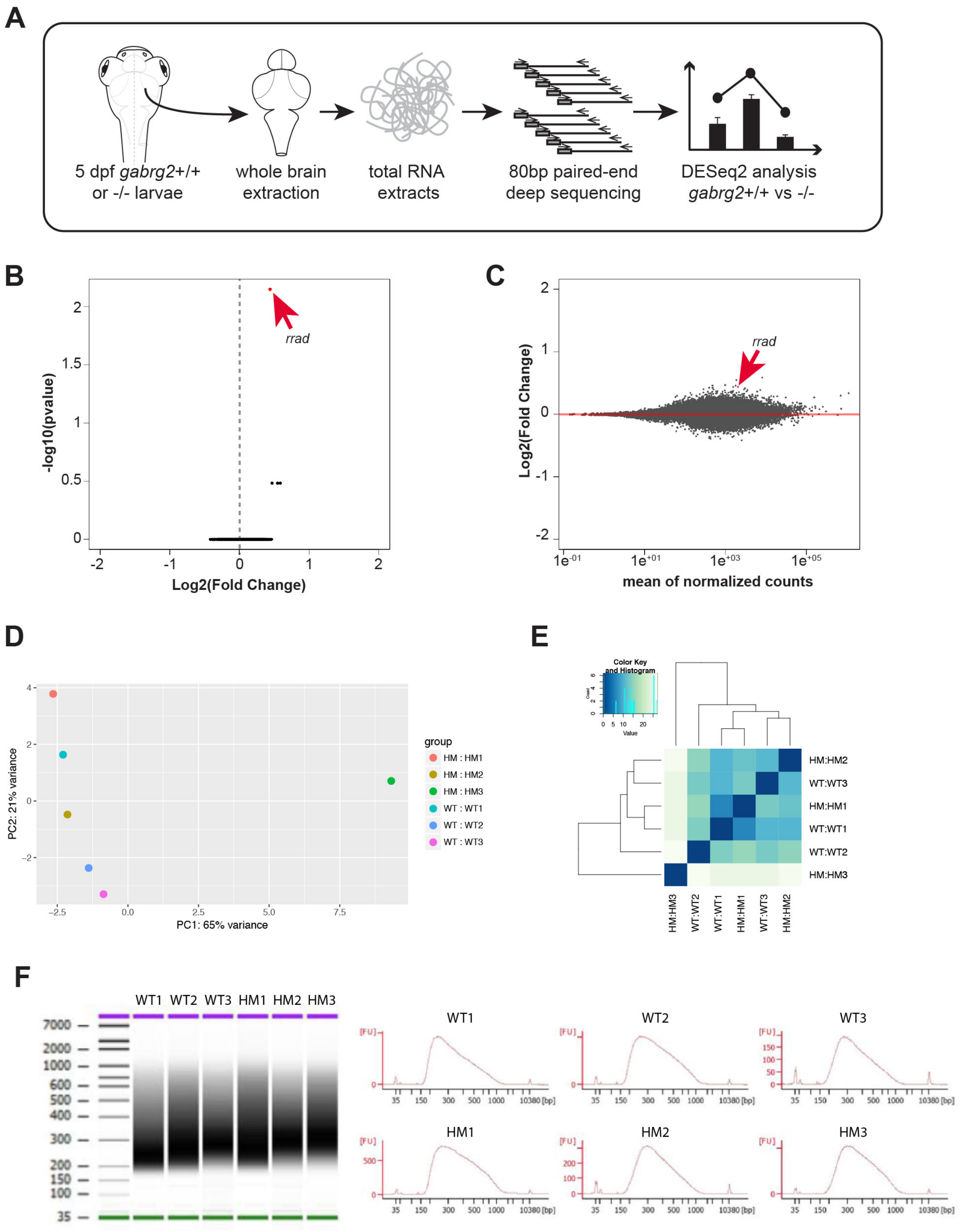

G

Alignment of exon 1 from gabrg2 gene in WT and HM samples

```
WT ATGGTGATGATGGCTTCTCTTCATTTTTCCTCTAAATGCCTGAA--C---GCGATGGCCATCCCCGCGGTGACACTCAAGCTCTTTCTGTGGGCTTTTCTAATCGCCCATCTTCCTCTGAG--------
HM ATGGTGATGATGGCTTCTCTTCATTTTTCCTCTAAATGCCTGAATGCGATGCGATGGCCATCCCCGCGGTGACACTCAAGCTCTTTCTGTGGGCTTTTCTAATCGCCCATCTTCCTCTGAG--------
```

Mutation coverage of the two variants in each samples

	Sample					
Variant	WT1	WT2	WT3	HM1	HM2	HM3
WT	53	87	74	5	0	0
Mutant	0	0	0	44	38	0

Disease Models & Mechanisms • Supplementary information

Table S1: Differential expression analysis from whole transcriptome sequencing.

Click here to Download Table S1

Table S2: qPCR validation of GABA-related gene expression in gabrg2-/- larvae.

Click here to Download Table S2

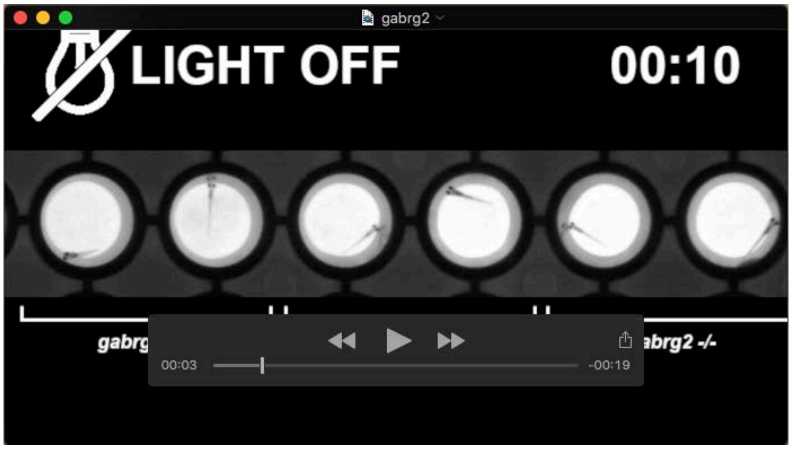

Movie 1: Light-induced reflex seizures in gabrg2-/- at 6 days-post-fertilization. 6 dpf larvae were accommodated individually in 96 wells for at least 30 minutes in a light-proof recording chamber (DanioVision, Noldus). The light was suddenly turned on (see countdown in movie).

Disease Models & Mechanisms • Supplementary information
